# Exosomes derived from Piwil2-induced cancer stem cells transform fibroblasts into cancer-associated fibroblasts

**DOI:** 10.3892/or.2022.8273

**Published:** 2022-01-27

**Authors:** Dan Zhang, Dian Li, Lianju Shen, Dong Hu, Bo Tang, Wenhao Guo, Zhang Wang, Zhaoxia Zhang, Guanghui Wei, Dawei He

Oncol Rep 43: 1125-1132, 2020; DOI: 10.3892/or.2020.7496

Following the publication of the above paper, the authors have realized that they made an error during the assembly of the western blotting data in Fig. 4; essentially, the western blotting data shown for the FAP experiment in Fig. 5 were mistakenly inserted into Fig. 4 to show the metalloproteinase-9 (MMP-9) data.

The authors re-examined their original data, and discovered how the error in the compilation of Fig. 4 arose. The corrected version of Fig. 4, incorporating the correct data for the MMP-9 experiment, is shown below. Note that this error did not affect the overall conclusions reported in the study. The authors are grateful to the Editor of *Oncology Reports* for allowing them the opportunity to publish this Corrigendum; furthermore, they apologize for any inconvenience caused to the readership of the Journal.

## Figures and Tables

**Figure 4.**
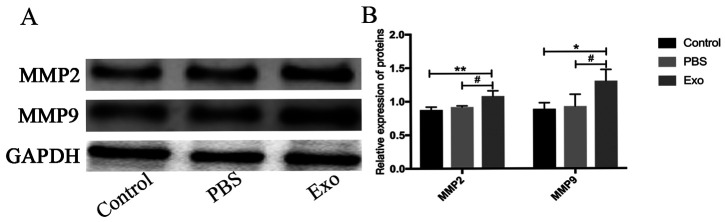
MMP2 and MMP9 expression levels in FBs following incubation with Piwil2-iCSC-Exo (200 µg/ml). GAPDH was used as a loading control. At least three independent experiments were conducted. Results were considered statistically significant at P<0.05 (*P<0.05, **P<0.01 vs. control group; ^#^P<0.05 vs. PBS group). One-way ANOVA and the LSD post hoc test were selected for analysis. FBs, fibroblasts; Piwil2-iCSC-Exo, exosomes derived from Piwil2-induced cancer stem cells; MMP, matrix metalloproteinase.

